# Singularity formation in 3D Euler equations with smooth initial data and boundary

**DOI:** 10.1073/pnas.2500940122

**Published:** 2025-06-27

**Authors:** Jiajie Chen, Thomas Y. Hou

**Affiliations:** ^a^Courant Institute of Mathematical Sciences, New York University, New York, NY 10012; ^b^Department of Computing and Mathematical Sciences, Caltech, Pasadena, CA 91125

**Keywords:** Euler equations, singularity formation, computer-assisted proof, fluid dynamics

## Abstract

A long-standing fundamental open problem in mathematical fluid dynamics and nonlinear partial differential equations is to determine whether solutions of the 3D incompressible Euler equations can develop a finite-time singularity from smooth, finite-energy initial data. Leonhard Euler introduced these equations in 1757 [L. Euler, *Mémoires de l’Académie des Sci. de Berlin* **11**, 274–315 (1757).], and they are closely linked to the Navier–Stokes equations and turbulence. While the general singularity formation problem remains unresolved, we review a recent computer-assisted proof of finite-time, nearly self-similar blowup for the 2D Boussinesq and 3D axisymmetric Euler equations in a smooth bounded domain with smooth initial data. The proof introduces a framework for (nearly) self-similar blowup, demonstrating the nonlinear stability of an approximate self-similar profile constructed numerically via the dynamical rescaling formulation.

## Significance

The problem of singularity formation of the 3D incompressible Euler equations with smooth initial data has remained open since Leonhard Euler introduced the equations in 1757. Beyond its importance in partial differential equations (PDE) analysis, the problem has drawn interest from physicists and engineers due to the potential impact of singularities on fluid flow modeling. This paper provides a rigorous solution of the problem for flows in a domain with a smooth boundary. It introduces a framework for studying nearly self-similar blowup, advancing our understanding of singularity formation not only in fluid mechanics but also in broader classes of nonlinear PDEs.

The three-dimensional (3D) incompressible Euler equations govern the motion of ideal incompressible fluids without external forcing in the inviscid setting. In the vorticity formulation, the Euler equations read

[1E] $$\begin{array}{c} \omega_{t}+u\cdot \nabla \omega =\omega \cdot \nabla u.\;u=\nabla \times {\left(-\Delta \right)}^{-1}\omega , \end{array}$$

where ω=∇×u is the vorticity vector and **u** is the velocity vector. These equations are among the most fundamental nonlinear partial differential equations (PDEs for short) with many important applications in science and engineering. Considerable effort has been devoted to studying the regularity properties of the 3D Euler equations; see, e.g., refs. 1 and 2. However, the fundamental questions concerning the global existence and regularity of solutions to these equations remain open and are widely regarded as among the most important in mathematical fluid mechanics.

**Fig. 1.**
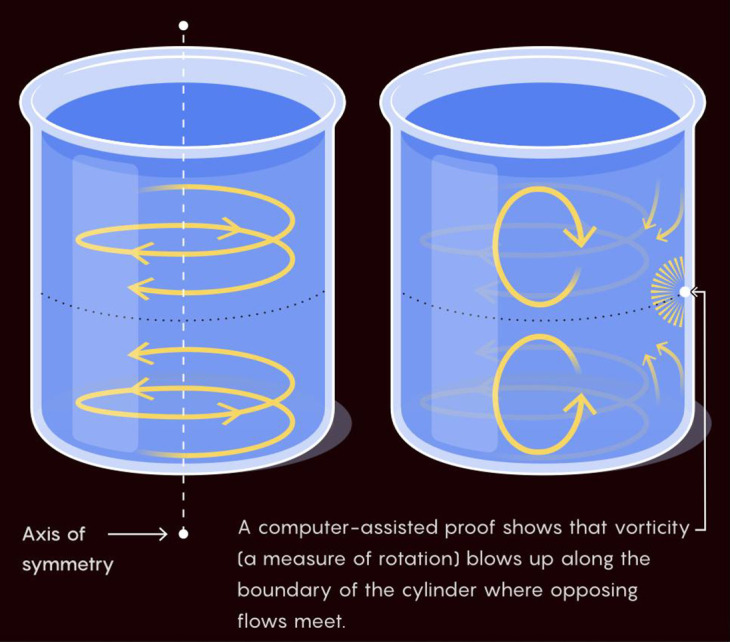
An illustration of the flow structure in the Hou–Luo blowup scenario. Image Credit: Merrill Sherman/Quanta Magazine. Reprinted with permission from ref. 5.

Numerical simulations by Hou and Luo provide strong evidence for a finite-time singularity in the 3D axisymmetric Euler equations on the boundary of a periodic cylinder (3, 4). The singularity occurs at the intersection of the boundary *r* = 1 and the symmetry plane *z* = 0 (Fig. 1). Axisymmetry, the odd symmetry in the *z* direction, and the presence of the boundary all contribute to this blowup.

As illustrated in Fig. 1, the flow forms an antisymmetric pattern, with *Top* and *Bottom* swirls driving azimuthal flow toward the critical ring at r=1,z=0. This generates intense vorticity, disrupting the geometric regularity of the vorticity vector near *z* = 0 (6, 7), and leads to singularity formation at a hyperbolic saddle point (8). Several related studies (9–14) further support the Hou–Luo scenario as a strong candidate for an Euler singularity.

Recently, we have rigorously justified the Hou–Luo blowup scenario and established the following blowup result, summarized from refs. 15 and 16.

Theorem 1. *There exists a family of smooth initial data with finite energy such that the solution of the 3D axisymmetric Euler equations Eq.* ***1E*** *in the cylinder* r,z∈[0,1]×T *develops a nearly self-similar singularity in finite time* *T*, *where* T *denotes the periodic torus*.

We develop a framework for (nearly) self-similar blowup by proving the stability of an approximate self-similar profile. Since the two-part paper is highly technical, we provide a high-level overview of the main ideas presented in refs. 15 and 16.

We remark that Elgindi and coauthors have proved a remarkable result that the 3D axisymmetric Euler equations in $R^{3}$ with $C^{1,\alpha }$ velocity, for small *α*, and without swirl develop a finite-time singularity (17, 18). The weak regularity class with very small *α* is essential in refs. 17 and 18. It is well known that the axisymmetric Euler equations without swirl have global regularity for smooth initial data (1). After our works (15, 16), finite-time blowup with $C^{1,\alpha }$ velocity and small *α* was constructed in ref. 19 for a new scenario and in ref. 20 using an iterative construction. Blowup with a solution-dependent force $f\in C^{1/2-}$ was established in ref. 21.

## 1.1. Difficulties.

There are several difficulties in analyzing blowup in the 3D Euler equations with smooth data. First, advection induces a stabilizing effect that can deplete the singularity (22, 23). Second, the velocity $u=\nabla \times {\left(-\Delta \right)}^{-1}\omega$ in Eq. **IE** depends nonlocally on the solution and is difficult to control. Third, the solution has a generic multidimensional structure.

In some previous works (13, 17, 24–26), the leading-order blowup profile can be reduced to a one-dimensional profile by imposing certain symmetry conditions. However, for the 3D Euler equations with smooth data, the incompressibility condition prevents such a reduction to a 1D PDE with small perturbations using natural symmetries. Thus, we must develop a new analytical method capable of handling the fully multidimensional problem.

## 1.2. Boundary.

In the Hou–Luo scenario, the potential singularity forms at a stagnation point, where the boundary mitigates the effect of advection. Certain sign and symmetry properties generate a hyperbolic flow near the blowup point and enhance vortex stretching. Although advection normal to the boundary pushes fluid away-suppressing growth near the singularity-this effect vanishes at the boundary due to the no-flow condition. Thus, vortex stretching induces significant growth of vorticity near the boundary. This mechanism is captured by the Hou–Luo model along the boundary (27). In contrast, in a similar setting for 2D Boussinesq without a boundary, vorticity with odd symmetry in both *x* and *y* vanishes on *x* = 0 and *y* = 0, weakening vortex stretching relative to advection and thereby destroying the blowup mechanism.

## 2. A Framework for Nearly Self-Similar Blowup

To introduce our framework, we consider a general nonlinear PDE for a scalar function *f*

[1] $$\begin{array}{c} f_{t}=N\left(f\right), \end{array}$$

where N(f) is a quadratic nonlinearity, such as *f*^2^ or $f\partial_{\mathrm{ij}}{\left(-\Delta \right)}^{-1}f$ for some i,j∈{1,2,3}, mimicking the vortex stretching term ω·∇u in Eq. **1E**.

We study a (nearly) self-similar blowup solution *f* to Eq. **1**, meaning that the solution maintains a similar pattern under suitable spatial rescaling and amplitude normalization. It can be described by:^*^

[2] $$\begin{array}{c} \begin{array}{c} f\left(t,x\right)\approx \frac{1}{T-t}F\left(t,\frac{x}{{\left(T-t\right)}^{\mu (t)}}\right),\\ ||F\left(t,\cdot \right)-\bar{F}{\left|\right|}_{X}\ll 1\;, \end{array} \end{array}$$

with some μ(t)>0, where F(t,·) remains close to an (approximate) blowup profile $\overset{¯}{F}\neq 0$ in some norm $\left|\right|\cdot {\left|\right|}_{X}$ uniformly up to the blowup time *T*.

To derive the equation for the rescaled solution *F*, we employ the dynamic rescaling formulation (28); see also ref. 29 for the related modulation technique. This approach dynamically and continuously rescales the physical solution both in space and time and in amplitude, according to certain normalization conditions. It yields an equivalent equation for *F* as follows

[3] $$\begin{array}{c} \partial_{\tau }F+c_{l}\left(\tau \right)x\cdot \nabla F=N\left(F\right)+c_{\omega }\left(\tau \right)F, \end{array}$$

where the parameter $c_{l}\left(\tau \right)$ is used to zoom in on the blowup region, while $c_{\omega }\left(\tau \right)$ rescales the amplitude of the solution. The exponent *μ* in Eq. **2** is primarily determined by $|c_{l}\left(\tau \right)/c_{\omega }\left(\tau \right)|$. We can freely choose $c_{l}\left(\tau \right)$ and $c_{\omega }\left(\tau \right)$ to define Eq. **3**. An (approximate) steady state $\overset{¯}{F}$ of Eq. **3** corresponds to an (approximate) blowup profile $\overset{¯}{F}$ in Eq. **2**. Moreover, the blowup time *t* = *T* maps to *τ* = *∞* in the rescaled time. See Section 2.1.1 for the full transform in the case of the Boussinesq equations.

Our framework consists of three steps:

1. Construct the approximate steady state and blowup exponents $\overset{¯}{G}=(\overset{¯}{F},{\overset{¯}{c}}_{l},{\overset{¯}{c}}_{\omega })$ to Eq. **3**;
2. Linearize the equations around $\left(\overset{¯}{F},{\overset{¯}{c}}_{l},{\overset{¯}{c}}_{\omega }\right)$ to obtain the perturbation equation for $\tilde{G}=\left(F\left(\tau \right)-\overset{¯}{F},c_{l}\left(\tau \right)-{\overset{¯}{c}}_{l},c_{\omega }\left(\tau \right)-{\overset{¯}{c}}_{\omega }\right)$:
  [4] $$\begin{array}{c} \partial_{\tau }\tilde{G}=L\tilde{G}+N\left(\tilde{G}\right)+R\left(\overset{¯}{G}\right), \end{array}$$
  where L,N,R are the linear terms, nonlinear terms, and residual error, respectively. We then establish nonlinear stability estimates for a certain energy E(τ) of the perturbation $\tilde{G}$, such as
  [5a] $$\begin{array}{c} \frac{d}{d\tau }E\left(\tau \right)\leq -\lambda E\left(\tau \right)+CE{\left(\tau \right)}^{2}+\varepsilon ,\;\lambda >0, \end{array}$$
  where −λE, $CE^{2}$, and *ε* estimate the contributions from the linear term, nonlinear term, and the residual error of the approximate steady state $\overset{¯}{F}$, respectively. Using a bootstrap argument, we can obtain nonlinear stability $E\left(\tau \right)<E^{*}$ for some threshold $E^{*}\ll 1$ if *ε* is sufficiently small. For example, the condition
  [5b] $$\begin{array}{c} \varepsilon <\lambda^{2}/\left(4C\right) \end{array}$$
  is sufficient to close Eq. **5**. This justifies $||F-{\overset{¯}{F}||}_{X}\ll 1$ in the ansatz Eq. **2**.
3. Construct the initial data with desired properties by perturbing the profile $\overset{¯}{F}$. Then, we construct a nearly self-similar blowup solution to Eq. **1** using the transform between Eq. **1** and Eq. **3**. Note that to prove blowup, we do not need to show that the solution *F* to Eq. **3** converges to an exact profile.

We first developed the above framework with Huang in refs. 27 and 30 for some 1D models of 3D Euler equations.

### 2.1. Dependency Tree of the Proof.

*Theorem* 1 is proved using nonlinear stability estimates and inequalities similar to Eq. **5**, which rely on the three main components in the dependency tree. The most difficult part is establishing linear stability, i.e., *λ* > 0 in Eq. **5a**. We will discuss in some detail the key ideas behind sharp Hölder estimates in Section 3.4.2, finite-rank perturbations in Section 3.5.1, and linear energy estimates in Sections 3.1.1 and 3.2.1. In addition, we will briefly outline the approaches for estimating nonlocal terms in Section 4.3, nonlinear estimates in Section 3.7.1, constructing the profile in Section 4.1, estimating the profile and residual error in Section 4.2, and performing computer-assisted estimates (not shown in the dependency tree) in Section 3.7.2.


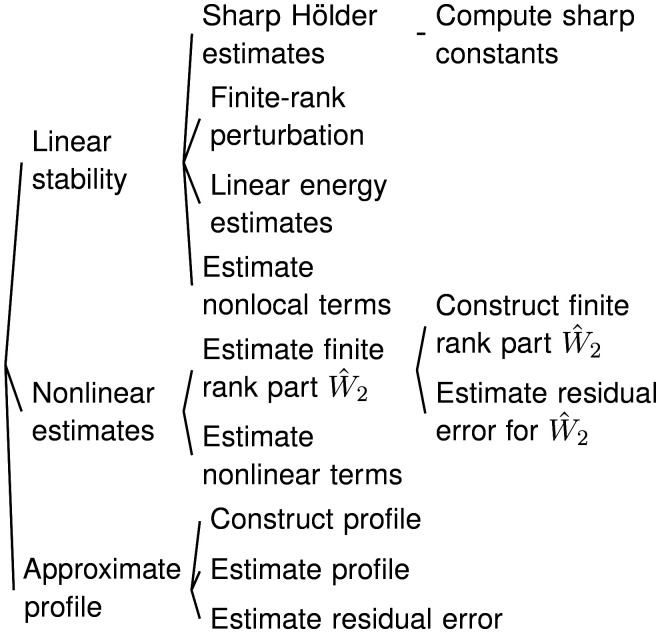


We refer to the more comprehensive dependency tree in 16, section 2.3, which illustrates how each part of the analysis maps to specific sections in refs. 15 and 16.

#### 2.1.1. Dynamic rescaling formulation.

The 3D axisymmetric Euler equations near the singularity (3) (located on the boundary away from the symmetry axis) can be formally approximated by the 2D Boussinesq (1, 13):

[6a] $$\partial_{t}\omega +u\cdot \nabla \omega =\theta_{x},$$

[6b] $$\partial_{t}\theta +u\cdot \nabla \theta =0,\;u=\nabla^{⊥}{\left(-\text{Δ}\right)}^{-1}\omega ,$$

in the upper half-space $\left(x,y\right)\in R_{+}^{2}$ with the no-flow boundary condition v(x,0)=0, where u=(u,v) is the velocity, $\omega \;=\;u_{y}-v_{x}$ is the vorticity, and *θ* is the density or temperature. It is easy to show that

$$\begin{array}{cc} \tilde{\omega }\left(\tau ,x\right) & =C_{\omega }\left(\tau \right)\omega \left(t\left(\tau \right),C_{l}\left(\tau \right)x\right),\\ \tilde{\theta }\left(\tau ,x\right) & =C_{\omega }^{2}\left(\tau \right)C_{l}{\left(\tau \right)}^{-1}\theta \left(t\left(\tau \right),C_{l}\left(\tau \right)x\right), \end{array}$$

solve the dynamic rescaling equations

[7] $$\begin{array}{c} \begin{array}{cc} {\tilde{\omega }}_{\tau }+\left(c_{l}\left(\tau \right)x+\tilde{u}\right)\cdot \nabla \tilde{\omega } & =c_{\omega }\left(\tau \right)\tilde{\omega }+{\tilde{\theta }}_{x},\\ {\tilde{\theta }}_{\tau }+\left(c_{l}\left(\tau \right)x+\tilde{u}\right)\cdot \nabla \tilde{\theta } & =\left(c_{l}\left(\tau \right)+2c_{\omega }\left(\tau \right)\right)\tilde{\theta }, \end{array} \end{array}$$

where $\tilde{u}={\left(\tilde{u},\tilde{v}\right)}^{T}=\nabla^{⊥}{\left(-\Delta \right)}^{-1}\tilde{\omega }$, $x={\left(x,y\right)}^{T}$, *τ* is the rescaled time, $c_{l}\left(\tau \right),c_{\omega }\left(\tau \right)$ are time-dependent scaling parameters, and $t\left(\tau \right)=\int_{0}^{\tau }C_{\omega }\left(\tau \right)d\tau$,

[8] $$\begin{array}{c} \begin{array}{cc} C_{\omega }\left(\tau \right) & =e^{\int_{0}^{\tau }c_{\omega }\left(s\right)d\tau },\;C_{l}\left(\tau \right)=e^{\int_{0}^{\tau }-c_{l}\left(s\right)ds}. \end{array} \end{array}$$

Eq. **7** corresponds to Eq. **3**. To simplify our presentation, we still use *t* to denote the rescaled time *τ* and simplify $\tilde{\omega },\tilde{\theta }$ as *ω*, *θ*. Eq. **7** becomes


[9] $$\begin{array}{c} \begin{array}{cc} \omega_{t}+\left(c_{l}x+u\right)\cdot \nabla \omega & =\theta_{x}+c_{\omega }\omega ,\\ \theta_{t}+\left(c_{l}x+u\right)\cdot \nabla \theta & =(c_{l}+2c_{\omega })\theta . \end{array} \end{array}$$

We impose normalization conditions on $c_{\omega },c_{l}$:

[10] $$\begin{array}{c} c_{l}\left(t\right)=2\frac{\theta_{\mathrm{xx}}\left(t,0\right)}{\omega_{x}\left(t,0\right)},\;c_{\omega }\left(t\right)=\frac{1}{2}c_{l}\left(t\right)+u_{x}\left(t,0\right). \end{array}$$

For smooth data, these two conditions enforce

[11] $$\begin{array}{c} \theta_{\mathrm{xx}}\left(t,0\right)=\theta_{0,xx}\left(0\right),\;\omega_{x}\left(t,0\right)=\omega_{0,x}\left(0\right), \end{array}$$

for all time. We close the Eqs. **9** and **10**.

Following the blowup framework in Section 2, we aim to construct the approximate blowup profile $\left(\overset{¯}{\omega },\overset{¯}{\theta },{\overset{¯}{c}}_{\omega },{\overset{¯}{c}}_{l}\right)$ for Eq. **9** and show that the perturbation $\tilde{G}=\left(\omega \left(t\right)-\overset{¯}{\omega },\theta \left(t\right)-\overset{¯}{\theta },c_{\omega }\left(t\right)-{\overset{¯}{c}}_{\omega },c_{l}\left(t\right)-{\overset{¯}{c}}_{l}\right)$ remains small in some energy functional E(·): $E(\tilde{G}\left(t\right))\ll 1$ for any *t* ≥ 0. With this stability estimate and the values of the approximate scaling parameters ${\overset{¯}{c}}_{l},{\overset{¯}{c}}_{\omega }$, the blowup asymptotics of the vorticity follow the form Eq. **2** with

$$F\approx \overset{¯}{\omega },\;\mu (t)\approx 2.92.$$

## 3. Key Methods in the Stability Estimates

The most difficult step is establishing the linear stability of the approximate profile. While refining the computation can yield a small residual error, e.g., *ε* in Eq. **5**, *ε* is not an arbitrarily small parameter, and obtaining a very small *ε* is highly challenging. To close the nonlinear estimates Eq. **5**, we must therefore develop quantitative and effective stability estimates with computable constants (e.g., *λ*, *C* in Eq. **5**).

A major challenge lies in controlling a compact operator arising from the nonlocal terms. A key distinction between our quantitative estimates of this compact operator (15, 16) (Section 3.5.1) and existing methods-such as finite codimension stability estimates (24, 31–34)-is that the latter rely on functional analysis and compactness arguments, resulting in implicit, uncomputable constants that cannot be used to close the argument in Eq. **5b**. In refs. 24 and 31–34, exact self-similar profiles (corresponding to *ε* = 0 in Eq. **5**) are constructed so that one does not need computable constants to close nonlinear estimates similar to Eq. **5b**.

### 3.1. Linearized Equations.

Linearizing Eq. **9** around the approximate steady state and neglecting nonlinear and residual error terms yield the linearized equations for the perturbation $\left(\tilde{\omega },\eta ,\xi \right)\equiv \left(\omega -\overset{¯}{\omega },\theta_{x}-{\overset{¯}{\theta }}_{x},\theta_{y}-{\overset{¯}{\theta }}_{y}\right)$

[12a] $$\begin{array}{l} \partial_{t}\omega +({\bar{c}}_{l}x+\bar{u})\cdot \nabla \omega =\eta +{\bar{c}}_{\omega }\omega \\ \;\;-u\cdot \nabla \bar{\omega }+c_{\omega }\bar{\omega }, \end{array}$$

[12b] $$\begin{array}{l} \partial_{t}\eta +({\bar{c}}_{l}x+\bar{u})\;\cdot \;\nabla \eta =(2{\bar{c}}_{\omega }-{\bar{u}}_{x})\eta -{\bar{v}}_{x}\xi \\ \;\;-u_{x}\;\cdot \;\nabla \bar{\theta }-u\;\cdot \;\nabla {\bar{\theta }}_{x}+2c_{\omega }{\bar{\theta }}_{x}, \end{array}$$

[12c] $$\begin{array}{l} \partial_{t}\xi +({\bar{c}}_{l}x+\bar{u})\;\cdot \;\;\nabla \xi =(2{\bar{c}}_{\omega }+{\bar{u}}_{x})\xi -{\bar{u}}_{y}\eta \\ \;\;-u_{y}\;\cdot \;\nabla \bar{\theta }-u\;\cdot \;\nabla {\bar{\theta }}_{y}+2c_{\omega }{\bar{\theta }}_{y}, \end{array}$$

where $u=\left(u,v\right)=\nabla^{⊥}{\left(-\Delta \right)}^{-1}\omega$, and $c_{\omega }=u_{x}\left(t,0\right)$ by linearizing Eqs. **10** and **11**. Here, we abuse the notation *ω* to represent the perturbation $\tilde{\omega }=\omega -\overset{¯}{\omega }$.

#### 3.1.1. Properties of the profile.

The approximate profile we constructed satisfies the following properties with some constants $\kappa ,\mu ,\gamma ,M_{k}>0$.

(P1) Symmetries: $\overset{¯}{\theta }(x,y),\overset{¯}{v}(x,y)$ are even in *x*, $\overset{¯}{\omega }(x,y),\overset{¯}{u}(x,y)$ are odd in *x*, and $\nabla \overset{¯}{\theta }(0)=0$.
(P2) Sign properties and scaling:
  [13a] $$\mu ≜{\bar{c}}_{l}+{\bar{u}}_{x}(0)=2{\bar{c}}_{\omega }-{\bar{u}}_{x}(0)>0,$$
  [13b] $${\bar{u}}_{x}(0)\;\;<0,\;\;{\bar{c}}_{l}>0,\;\;\;{\bar{c}}_{\omega }\;<-0,\;-{\bar{c}}_{l}/{\bar{c}}_{\omega }>2.$$
(P3) Outflow flows: For any x,y≥0, we have
  [13c] $$\begin{array}{c} {\overset{¯}{c}}_{l}x+\overset{¯}{u}\left(x,y\right)\geq \kappa x,\;{\overset{¯}{c}}_{l}y+\overset{¯}{v}\left(x,y\right)\geq \kappa y; \end{array}$$
(P4) Decay conditions: For *k* ≤ 2, we have
  [13d] $$\begin{array}{c} \begin{array}{cc} |\nabla^{k}\overset{¯}{\omega }| & \leq M_{k}{\;\min(1,|x|}^{-\gamma -k}),\\ |\nabla^{k+1}\overset{¯}{\theta }| & \leq M_{k}{\;\min(1,|x|}^{-2\gamma -k}),\\ |\nabla^{k}\overset{¯}{u}| & \leq M_{k}{\;\min(1,|x|}^{1-\gamma -k}). \end{array} \end{array}$$

Let us explain property (P2). The condition ${\overset{¯}{c}}_{l}>0$ indicates that the blowup is focusing, while ${\overset{¯}{c}}_{\omega }<0$ implies finite-time blowup: t(∞)<∞ from Eq. **8**. Moreover, the condition $-{\overset{¯}{c}}_{l}/{\overset{¯}{c}}_{\omega }>2$ follows from the conservation of ${\left||\theta |\right|}_{L^{\infty }}$ in the 2D Boussinesq equations Eq. **6b**. The second identity in Eq. **13a** is a consequence of the normalization condition Eq. **10**.

### 3.2. Stability Mechanism.

The outflow property Eq. **13c** for the transport coefficients in Eq. **12** plays a crucial role in establishing stability; see *Lemma* 2. It transports perturbations from the origin x=0 to the far field. In addition, the decay condition Eq. **13d** and the sign condition ${\overset{¯}{c}}_{\omega }<0$ from Eq. **13a** imply that the system Eq. **12** is stable in the far field. These features form the core stability mechanisms of Eq. **12**. We use them to prove stability (i.e., *λ* > 0 in Eq. **5b**) for the operator $L_{0}=L-K_{n}$, where L is the linearized operator in Eq. **12** and $K_{n}$ is a finite-rank perturbation. The operator $K_{n}$ may introduce finitely many unstable eigenvalues to the full operator L.

We do not know a priori whether L Eq. **12** is fully stable or stable modulo finitely many unstable directions. Even for simpler equations, such as the Burgers equation (35) or semilinear heat equation, some blowup profiles exhibit full stability while others are stable up to finitely many unstable modes.

In the Hou–Luo scenario, it was shown in ref. 36, section 3.4 that a discretized version of the linearized operator in a suitable finite-dimensional basis possesses a spectral gap. Moreover, our numerical results suggest that the blowup profile is not sensitive to perturbations in the initial data, as long as the initial data satisfy certain sign and symmetry conditions. To extend stability from $L-K_{n}$ to the full operator L, we apply the approach in Section 3.5.1, which implicitly leverages these numerical observations.

#### 3.2.1. Stability of the linearized operator with finite-rank perturbation.

In this section, we discuss the method to show that the linearized operator Eq. **12** is stable in some suitable weighted space up to some finite-rank operator; see Eq. **25**.

To illustrate the key ideas for the stability estimates, we consider the following model problem

[14] $$\begin{array}{c} \partial_{t}f+\overset{¯}{V}\cdot \nabla f=\overset{¯}{a}\left(x\right)f+\overset{¯}{b}\left(x\right)B\left(f\right),\;\overset{¯}{V}={\overset{¯}{c}}_{l}x+\overset{¯}{u}, \end{array}$$

where $\nabla \cdot \overset{¯}{u}=0$ and $\overset{¯}{V}$ satisfies the sign and outflow properties Eqs. **13a**–**13c**

[15] $$\begin{array}{c} {\overset{¯}{u}}_{x}\left(0\right)<0,\;{\overset{¯}{c}}_{l}+{\overset{¯}{v}}_{y}\left(0\right)>{\overset{¯}{c}}_{l}+{\overset{¯}{u}}_{x}\left(0\right)=\mu >0. \end{array}$$

The definition of *μ* is consistent with Eq. **13a**. The term $\overset{¯}{a}(x)f$ captures the diagonal part on the right side of Eq. **12**: ${\overset{¯}{c}}_{\omega }\omega ,\left(2{\overset{¯}{c}}_{\omega }-{\overset{¯}{u}}_{x}\right)\eta$, or $(2{\overset{¯}{c}}_{\omega }+{\overset{¯}{u}}_{x})\xi$, and we consider three cases of $\left(f,\overset{¯}{a}\right)$:

[16] $$\begin{array}{c} \left(f,\overset{¯}{a}\right)=\left(\omega ,{\overset{¯}{c}}_{\omega }\right),\left(\eta ,2{\overset{¯}{c}}_{\omega }-{\overset{¯}{u}}_{x}\right),\;\text{or}\;\left(\xi ,2{\overset{¯}{c}}_{\omega }+{\overset{¯}{u}}_{x}\right). \end{array}$$

The coefficients $\overset{¯}{a}(x)$, $\overset{¯}{b}(x)$ satisfy the decay property

[17] $$\begin{array}{c} \begin{array}{cc} |\nabla^{k}\overset{¯}{b}\left(x\right)|,\;|\nabla^{k}\left(\overset{¯}{a}\left(x\right)-\overset{¯}{a}\left(\infty \right)\right)| & \leq M_{k}{\;\min(1,|x|}^{-\gamma -k}),\\ |\overset{¯}{b}(x)| & \leq M_{0}{\;\min(|x|,|x|}^{-\gamma }), \end{array} \end{array}$$

for *k* ≤ 2 with $\overset{¯}{a}\left(\infty \right)=\;\lim_{|x|\to \infty }\overset{¯}{a}\left(x\right)$, and $B\left(f\right)=K_{B}*f$ is a 0-th order singular integral operator related to the velocity, e.g. $B\left(f\right)=\partial_{\mathrm{ij}}{\left(-\Delta \right)}^{-1}f,\;K_{B}\left(z\right)=\partial_{\mathrm{ij}}\log\left(z\right)$ for i,j∈{1,2}. The nonlocal term $\overset{¯}{b}(x)B(f)$ models the leading order nonlocal terms $u_{x}\cdot \nabla \overset{¯}{\theta }$ and $u_{y}\cdot \nabla \overset{¯}{\theta }$ in Eqs. **12b** and **12c**, ^†^ where the coefficient $\nabla \overset{¯}{\theta }$ vanishes near the origin. Note that we do not require the coefficients to be small, nor do we assume that $\overset{¯}{a}$ is negative. To extract the damping part, we use the following weighted estimates:

[18] $$\begin{array}{c} \begin{array}{cc} & \partial_{t}\left(f\varphi \right)+\overset{¯}{V}\cdot \nabla \left(f\varphi \right)\\ & =(\overset{¯}{a}\left(x\right)+\frac{\overset{¯}{V}\cdot \nabla \varphi }{\varphi })\cdot \left(f\varphi \right)+\overset{¯}{b}\left(x\right)B\left(f\right)\varphi \\ & ≜d(\varphi )\cdot (f\varphi )+B, \end{array} \end{array}$$

with a singular weight *φ* to be determined.

##### 3.2.1.1. Outflow property and the damping terms.

We consider the following weight

[19a] $$\begin{array}{c} \varphi =\varphi_{s}{\hat{\varphi }}^{m},\;\varphi_{s}={\left|x\right|}^{\alpha }+{\left|x\right|}^{\beta }, \end{array}$$

where the singular weight *φ*_*s*_ with α<0,α<β captures the asymptotics of the weights, $\hat{\varphi }≍1$ is more regular, and *m* > 0. We choose *α*, *β* such that

[19b] $$\begin{array}{c} \overset{¯}{a}\left(0\right)+\mu \alpha \leq -\lambda ,\;\overset{¯}{a}\left(\infty \right)+{\overset{¯}{c}}_{l}\beta \leq -\lambda \;, \end{array}$$

for some *λ* > 0, where *μ* is defined in Eq. **15**, $\overset{¯}{a}$ in Eq. **16**, and $\overset{¯}{a}\left(\infty \right)=\;\lim_{|x|\to \infty }\overset{¯}{a}\left(x\right)$. See more discussions in Section 3.3.1 for this constraint on stability. We can use the outflow property Eq. **13c** and $\hat{\varphi }$ to generate arbitrarily large damping terms in a bounded region away from the origin.

Lemma 2. *Let* d(φ) *be the coefficient defined in* *Eq.* ***18***. *For any* $0<R_{1}<R_{2},D>0$ *and* $0<\lambda_{2}<\lambda$*,* *there exists smooth* $\hat{\varphi }≍1$ *and* *m* *> 0 depending on* $R_{1},R_{2},D,\lambda_{2}$ *such that*

$$d\left(\varphi \right)\leq -\lambda_{2}-D1_{R_{1}\leq \left|x\right|\leq R_{2}}.$$

The left side of Eq. **19b** captures the asymptotics of d(φ) near the origin and in the far-field, respectively.

***Proof:*** For fixed *λ*_2_ and *D*, we only need to prove the lemma for *R*_1_ sufficiently small and *R*_2_ large.

We represent the coefficient d(φ) in Eq. **18** as


[20] $$\begin{array}{c} \begin{array}{cc} & d\left(\varphi \right)=\overset{¯}{a}+\frac{\overset{¯}{V}\cdot \nabla \varphi }{\varphi }\\ & \;=m\frac{\overset{¯}{V}\cdot \nabla \hat{\varphi }}{\hat{\varphi }}+(\overset{¯}{a}\left(x\right)+\left(\overset{¯}{V}\cdot x\right)\cdot \frac{{\alpha |x|}^{\alpha -2}+\beta {\left|x\right|}^{\beta -2}}{{\left|x\right|}^{\alpha }+{\left|x\right|}^{\beta }})\\ & \;=J_{1}+J_{2}. \end{array} \end{array}$$

We estimate *J*_2_ for small |x| and large |x| differently. Near 0, using Eq. **13d** for $\overset{¯}{a}(x)$, we get

$$\begin{array}{cc} J_{2}\leq & a\left(0\right)+\overset{¯}{C}\;\min(|x{|}^{\beta -\alpha },|x|,1)\\ & +\left(\partial_{1}{\overset{¯}{V}}_{1}\left(0\right)x_{1}^{2}+\partial_{2}{\overset{¯}{V}}_{2}\left(0\right)x_{2}^{2}\right)\cdot \alpha {\left|x\right|}^{-2}, \end{array}$$

for some constant $\overset{¯}{C}$ depending on *α*, *β* in Eq. **19** and *M*_*k*_ Eq. **13d** but independent of *m*, which can vary from line to line.^‡^ Since *α* < 0 from Eq. **19** and $\partial_{1}{\overset{¯}{V}}_{1}\left(0\right),\partial_{2}{\overset{¯}{V}}_{2}\left(0\right)\geq \mu$ from Eqs. **14** and **15**, we get

$$\begin{array}{cc} J_{2} & \leq \overset{¯}{a}\left(0\right)+\mu \alpha +\overset{¯}{C}\;\min(|x{|}^{\beta -\alpha },|x|,1)\\ & \leq -\lambda +\overset{¯}{C}\min(|x{|}^{\beta -\alpha },|x|,1), \end{array}$$

where we have used Eq. **19** in the last inequality. Similarly, using the decay of $\overset{¯}{a}$ from Eqs. **13d** and **19**, we obtain

$$\begin{array}{cc} J_{2} & \leq a\left(\infty \right)+{\overset{¯}{c}}_{l}\beta +{\overset{¯}{C}\min(|x|}^{\alpha -\beta }{,|x|}^{-\beta },1)\\ & \leq -\lambda +\overset{¯}{C}\min(|x{|}^{\alpha -\beta },|x{|}^{-\beta },1). \end{array}$$

For $\lambda_{2}<\lambda$, optimizing the above estimates, there exists $0<{\overset{¯}{R}}_{1}\left(\lambda_{2}\right)<{\overset{¯}{R}}_{2}\left(\lambda_{2}\right)$ such that

[21] $$\begin{array}{c} J_{2}\leq -\lambda_{2}+\overset{¯}{C}1_{{\overset{¯}{R}}_{1}\leq \left|x\right|\leq {\overset{¯}{R}}_{2}}. \end{array}$$

Given any $R_{1},R_{2}>0$ with $R_{1}\leq {\overset{¯}{R}}_{1}\left(\lambda_{2}\right),{\overset{¯}{R}}_{2}\left(\lambda_{2}\right)\leq R_{2}$, we choose a radial weight $\hat{\varphi }$ that satisfies

$$\begin{array}{ccc} \hat{\varphi }\left(r\right) & =1,\;r\leq \frac{R_{1}}{2}, & \;\hat{\varphi }\left(r\right)=\frac{1}{2},\;r\geq 2R_{2},\\ \partial_{r}\hat{\varphi }\left(r\right) & \leq 0,\;r\geq 0,\; & \partial_{r}\hat{\varphi }\left(r\right)<0,\;R_{1}\leq r\leq R_{2}. \end{array}$$

It follows $\frac{r\partial_{r}\hat{\varphi }}{\hat{\varphi }}\leq -\lambda_{3}1_{R_{1}\leq r\leq R_{2}}$ for some $\lambda_{3}=\lambda_{3}\left(R_{1},R_{2}\right)>0$. Using this estimate and the outflow property Eq. **13c**, we obtain

$$\begin{array}{c} \overset{¯}{V}\left(x\right)\cdot x\geq {\kappa |x|}^{2}=\kappa r^{2},\\ J_{1}=m\frac{\overset{¯}{V}\cdot \nabla \hat{\psi }}{\hat{\psi }}=m\frac{\overset{¯}{V}\;\cdot x}{r^{2}}\cdot \frac{r\partial_{r}\hat{\psi }}{\hat{\psi }}\leq -m\lambda_{3}\kappa 1_{R_{1}\leq r\leq R_{2}}. \end{array}$$

Plugging the above estimates in *J*_1_ in Eq. **20**, and then using Eq. **21**, we prove

$$d\left(\varphi \right)\left(x\right)=J_{1}+J_{2}\leq -\lambda_{2}+\left(\overset{¯}{C}-m\lambda_{3}\kappa \right)1_{R_{1}\leq r\leq R_{2}}.$$

Choosing *m* large enough, we prove the lemma.

##### 3.2.1.2. Nonlocal part.

For the nonlocal part in Eq. **18**, we have

[22a] $$\begin{array}{c} B=\overset{¯}{b}\left(x\right)B\left(f\varphi \right)-\overset{¯}{b}\left(x\right)\left[B,\varphi \right]\left(f\right)=B_{M}\left(f\right)-B_{C}\left(f\right), \end{array}$$

where $B_{C}$ is the commutator

[22b] $$\begin{array}{c} \begin{array}{cc} B_{C}\left(f\right)\left(x\right) & =\overset{¯}{b}(x)(B(f\varphi )-\varphi B(f))\\ & =\overset{¯}{b}\left(x\right)\int K_{B}\left(x-y\right)(1-\frac{\varphi (x)}{\varphi (y)})\left(f\varphi \right)\left(y\right)dy. \end{array} \end{array}$$

A crucial observation is that the leading order nonlocal term $B_{M}\left(f\right)=\overset{¯}{b}\left(x\right)B\left(f\varphi \right)$ depends only on the weighted variable fφ. Therefore, it can be estimated with constants that are independent of the weight *φ*. Since the coefficient $\overset{¯}{b}(x)$ vanishes near x=0 and decays for large **x** Eq. **17**, we know that $\overset{¯}{b}(x)$ is small in both regions. By leveraging the strong damping term from *Lemma* 2 within a bounded region $x:|x|\in [R_{1},R_{2}]$ away from 0, we can treat $\overset{¯}{b}(x)B(f\varphi )$ as a small perturbation to the local damping term d(φ)·(fφ) in the $L^{\infty }$ energy estimate.^§^

Since $\overset{¯}{b}(x)$ vanishes near x=0 and decays in the far field by Eq. **17**, and the weight *φ* Eq. **19** is singular only near x=0, $1-\frac{\varphi (x)}{\varphi (y)}$ cancels one order of singularity in the kernel $K_{B}\left(x-y\right)$. This allows us to show that the operator $B_{C}$ is roughly one order more regular than *f*.^¶^ For instance, if $f\in L^{\infty }$, then $B_{C}\left(f\right)$ is log-Lipschitz in a suitable weighted space. Moreover, $B_{C}\left(f\right)$ can be approximated by a finite-rank operator.

### 3.3. Finite-Rank Operator.

One approach is to use a piecewise approximation

[23a] $$\begin{array}{c} K_{\mathrm{MN}}\left(f\right)\left(x\right)=\sum_{i\leq M,j\leq N}\chi_{\mathrm{ij}}\left(x\right)B_{C}\left(f\right)\left(x_{\mathrm{ij}}\right), \end{array}$$

where $\chi_{\mathrm{ij}}\left(x\right)\geq 0$ is some sufficiently smooth cutoff functions covering a domain $\left[0,R_{1}\right]\times \left[0,R_{2}\right]$

[23b] $$\begin{array}{c} \sum \chi_{\mathrm{ij}}\left(x\right)=1,\;x\in \left[0,R_{1}\right]\times \left[0,R_{2}\right], \end{array}$$

in the first quadrant and it is supported locally near $x_{\mathrm{ij}}$ with support size of order *h*, e.g., $x_{\mathrm{ij}}=\left(ih,jh\right)$ with $0\leq i\leq \frac{R_{1}}{h},0\leq j\leq \frac{R_{2}}{h}$. Each term $B_{C}\left(f\right)\left(x_{\mathrm{ij}}\right)$ is a rank-one operator and can be expressed as follows

$$\chi_{\mathrm{ij}}\left(x\right)B_{C}\left(f\right)\left(x_{\mathrm{ij}}\right)=p_{\mathrm{ij}}\left(x\right)\int fq_{\mathrm{ij}}dx,$$

for some function $p_{\mathrm{ij}},q_{\mathrm{ij}}$. Since *χ*_*ij*_ has a support size of O(h), we obtain using Eq. **23** that for $x\in \left[0,R_{1}\right]\times \left[0,R_{2}\right]$

$$\begin{array}{cc} & |K_{\mathrm{MN}}\left(f\right)\left(x\right)-B_{C}\left(f\right)\left(x\right)|\\ & =|\sum_{\mathrm{ij}}\chi_{\mathrm{ij}}\left(x\right)\left(B_{C}\left(f\right)\left(x\right)-B_{C}\left(f\right)\left(x_{\mathrm{ij}}\right)\right)|\\ & \leq Ch^{\alpha }{\left\|B_{C}\left(f\right)\right\|}_{{\dot{C}}^{\alpha }}\sum \chi_{\mathrm{ij}}\left(x\right), \end{array}$$

where we have used $|x-x_{\mathrm{ij}}|\leq Ch$ for $x\in \;\text{supp}(\chi_{\mathrm{ij}})$ in the inequality. Since $B_{C}\left(f\right)$ is about one order more regular than *f*, we further obtain

[24] $$\begin{array}{c} |K_{\mathrm{MN}}\left(f\right)\left(x\right)-B_{C}\left(f\right)\left(x\right)|\leq C_{\alpha }\left(\varphi \right)h^{\alpha }{\left\|f\varphi \right\|}_{L^{\infty }}, \end{array}$$

for any α∈(0,1), the constant $C_{\alpha }\left(\varphi \right)$ depends on the weight *φ* and *α*, but is independent of *h*. The approximation error is also small in the $C^{1/2}$ norm by choosing $\alpha >\frac{1}{2}$ and small *h*. For $x\notin \left[0,R_{1}\right]\times \left[0,R_{2}\right]$ with $R_{1},R_{2}$ suitably large, we gain smallness in the upper bound of Eq. **24** from the decay of $\overset{¯}{b}(x)$ in $B_{C}$; see Eq. **22b**. By choosing $R_{1},R_{2}$ and the rank *M*, *N* suitably large so that *h* is small, we can treat the approximation error $|K_{\mathrm{MN}}\left(f\right)\left(x\right)-B_{C}\left(f\right)\left(x\right)|$ perturbatively in the energy estimate.

Using the above method, we can establish exponential decay estimates of the linearized operator in some suitable weighted space up to a finite-rank operator

[25] $$\begin{array}{c} \partial_{t}f=\left(L-K_{\mathrm{MN}}\right)f. \end{array}$$

We will discuss more on the construction of $K_{\mathrm{MN}}$ and the estimate of $K_{\mathrm{MN}}$ in Section 3.5.1.

#### 3.3.1. Singularity and asymptotics of weights.

Below, we discuss the condition Eq. **19b** for the weighted estimates of ω,η,ξ in the linearized equations Eq. **12**. One may choose weights as

[26] $$\begin{array}{c} \varphi_{f}={\left(|x\right|}^{\alpha_{f}}+{\left|x\right|}^{\beta_{f}}){\hat{\varphi }}^{m},\;f=\omega ,\eta ,\xi . \end{array}$$

Due to the decay and vanishing conditions of the coefficients in the off-diagonal local part in Eq. **12**—e.g., ${\overset{¯}{v}}_{x}$ in ${\overset{¯}{v}}_{x}\xi$ and ${\overset{¯}{u}}_{y}$ in ${\overset{¯}{u}}_{y}\eta$—we can treat these terms perturbatively for |x| very small or very large. Recall the definition of $\overset{¯}{a}(x)$ from Eq. **16**. We have ${\overset{¯}{a}}_{\eta }\left(x\right)=2{\overset{¯}{c}}_{\omega }-{\overset{¯}{u}}_{x}$ in the *η*-equation and ${\overset{¯}{a}}_{\xi }=2{\overset{¯}{c}}_{\omega }+{\overset{¯}{u}}_{x}$ in the *ξ*-equation. Using *μ* from Eq. **13a** (same as Eq. **15**) and the decay of ${\overset{¯}{u}}_{x}$ from Eq. **13d**, we obtain

[27] $$\begin{array}{c} \begin{array}{ccc} & {\overset{¯}{a}}_{\eta }\left(0\right)=\mu ,\; & {\overset{¯}{a}}_{\eta }\left(\infty \right)=2{\overset{¯}{c}}_{\omega },\\ & {\overset{¯}{a}}_{\xi }\left(0\right)=4{\overset{¯}{c}}_{\omega }-\mu <0,\; & {\overset{¯}{a}}_{\xi }\left(\infty \right)=2{\overset{¯}{c}}_{\omega }. \end{array} \end{array}$$

Plugging Eq. **27** into Eq. **19b**, to design the weight *φ*_*η*_ for estimating $\eta \varphi_{\eta }$, we require

[28a] $$\begin{array}{c} \begin{array}{ccc} & \mu +\mu \alpha_{\eta }<0,\; & 2{\overset{¯}{c}}_{\omega }+{\overset{¯}{c}}_{l}\beta_{\eta }<0,\\ \Rightarrow \; & \alpha_{\eta }<-1,\; & \beta_{\eta }<-2{\overset{¯}{c}}_{\omega }/{\overset{¯}{c}}_{l}. \end{array} \end{array}$$

Similarly, to design the weight *φ*_*ξ*_ for $\xi \varphi_{\xi }$, we require

[28b] $$\begin{array}{c} \begin{array}{ccc} & 4{\overset{¯}{c}}_{\omega }-\mu +\mu \alpha_{\xi }<0,\; & 2{\overset{¯}{c}}_{\omega }+{\overset{¯}{c}}_{l}\beta_{\xi }<0,\\ \Rightarrow \; & \alpha_{\xi }<1-4{\overset{¯}{c}}_{\omega }/\mu ,\; & \beta_{\xi }<-2{\overset{¯}{c}}_{\omega }/{\overset{¯}{c}}_{l}. \end{array} \end{array}$$

The variable *ξ* admits much better stability estimates. Recall the normalization conditions Eq. **11** on *θ*, which imply $\eta =O(|x{|}^{2})$ and $\xi =O(|x{|}^{2})$ near x=0. Thus, we can choose $a_{\eta }\leq -2$ and $a_{\xi }<0$ to satisfy the first condition in Eq. **19b**. Since $-\frac{2{\overset{¯}{c}}_{\omega }}{c_{l}}>0$, we can select $\beta_{\eta },\beta_{\xi }\in \left(0,-\frac{2{\overset{¯}{c}}_{\omega }}{c_{l}}\right)$ to satisfy the second condition in Eq. **19b** and to capture the decay of *η* and *ξ* using these weights.

Although the coefficient 1 in the off-diagonal local term *η* in the *ω*-equation Eq. **12a** does not decay, we can still treat *η* perturbatively for |x| very small or very large by designing *φ*_*ω*_ in Eq. **26** for the *ω*-equation Eq. **12a** to be weaker than *φ*_*η*_. We choose

[28c] $$\begin{array}{c} \alpha_{\omega }-\alpha_{\eta }>0,\;\beta_{\omega }-\beta_{\eta }<0. \end{array}$$

Then the coefficient in the estimate

$$|\eta \varphi_{\omega }|\leq \frac{\varphi_{\omega }}{\varphi_{\eta }}|\eta \varphi_{\eta }{\left|≲\min(|x\right|}^{\alpha_{\omega }-\alpha_{\eta }}{,|x|}^{\beta_{\omega }-\beta_{\eta }})|\eta \varphi_{\eta }|$$

is small for |x| very small or very large.

We now return to the constraint Eq. **19b**. For *ω*-equation Eq. **12a**, we have ${\overset{¯}{a}}_{\omega }\left(x\right)={\overset{¯}{c}}_{\omega }$ and require

[28d] $$\begin{array}{c} \begin{array}{ccc} & {\overset{¯}{c}}_{\omega }+\mu \alpha_{\omega }<0,\; & {\overset{¯}{c}}_{\omega }+{\overset{¯}{c}}_{l}\beta_{\omega }<0,\\ \Rightarrow \; & \alpha_{\omega }<-{\overset{¯}{c}}_{\omega }/\mu ,\; & \beta_{\omega }<-{\overset{¯}{c}}_{\omega }/c_{l}. \end{array} \end{array}$$

Note that the upper bound for *β*_*ω*_ is smaller than that for $\beta_{\eta },\beta_{\xi }$, as given by Eqs. **28a** and **28b**. This indicates that we can choose $\varphi_{\eta },\varphi_{\xi }$ with slower decay than *φ*_*ω*_ and obtain faster decay estimates for *η*, *ξ* than for *ω*. The first condition in Eq. **28d** holds for any $\alpha_{\omega }<0$. It is straightforward to verify that the conditions in Eq. **28** hold for a wide range of $\alpha_{\cdot },\beta_{\cdot }$ with $\alpha_{\cdot }\geq -3$.

### 3.4. Same Velocity.

In Eq. **12**, the velocity ${\overset{¯}{c}}_{l}x+\overset{¯}{u}$ is the same for all three equations. Thus, we can extract the outflow property using the same factor $\hat{\varphi }$ in Eq. **26**.

#### 3.4.1. Weighted estimates in ref. 15.

For the blowup profile, we have

$$-{\overset{¯}{c}}_{\omega }/{\overset{¯}{c}}_{l}\approx 0.34.$$

In ref. 15, we first perform weighted $L^{\infty }$ estimates with faster decay weights (see *φ*_*i*_ in ref. 15, appendix C)

[29a] $$\begin{array}{c} \alpha_{\omega ,\eta ,\xi }=\left(-2.9,-3,-3\right),\;\beta_{\omega ,\eta ,\xi }=\left(-\frac{1}{6},\frac{1}{7},\frac{1}{7}\right), \end{array}$$

where $\alpha_{\omega ,\eta ,\xi }=\left(\alpha_{\omega },\alpha_{\eta },\alpha_{\xi }\right)$ and $\beta_{\omega ,\eta ,\xi }=\left(\beta_{\omega },\beta_{\eta },\beta_{\xi }\right)$. We use a similar notation below in Eq. **29b**. To further bound ω,η,ξ in $L^{\infty }$, we perform additional weighted $L^{\infty }$ estimates with growing weights (see $\varphi_{g,i},i=1,2,3$ in ref. 15, appendix C)

[29b] $$\begin{array}{c} \begin{array}{cc} \alpha_{\omega ,\eta ,\xi }^{\text{gr}} & =(-2.9,-3,-3),\\ \beta_{\omega ,\eta ,\xi }^{\text{gr}} & =(\frac{1}{16},\frac{1}{3}+10^{-8},\frac{1}{3}+10^{-8}). \end{array} \end{array}$$

The exponents in Eqs. **29a** and **29b** satisfy conditions Eq. **28** for stability. In ref. 15, instead of designing the weight using the formula from Eq. **19**, we use

[29c] $$\begin{array}{c} \varphi =\sum_{i\leq n}p_{i}{\left|x\right|}^{\alpha_{i}},\;\text{or}\varphi =|x_{1}{|}^{-1/2}\sum_{i\leq n}p_{i}{\left|x\right|}^{\alpha_{i}}. \end{array}$$

That is, we use a power function ${\left|x\right|}^{\alpha_{i}}$ instead of ${\hat{\varphi }}^{m}$ for the regular part. These choices of weights allow us to obtain a stronger weight in the far-field and obtain better embedding constants (smaller *C* in Eq. **5**), e.g., $\left|\omega \right|\leq \varphi^{-1}{\left||\omega \varphi |\right|}_{L^{\infty }}$, to close the nonlinear estimates. Moreover, the explicit form in Eq. **29c** makes it easier to estimate the piecewise bounds of the weights.

#### 3.4.2. Weighted C^1/2^ estimates and optimal transport.

Since the Riesz transform $\partial_{\mathrm{ij}}{\left(-\Delta \right)}^{-1}$ associated with the nonlocal term (see the discussion below Eq. **17** is not bounded in $L^{\infty }$, we perform the weighted $C^{1/2}$ estimates in ref. 15 to close the estimates. We use ideas and methods similar to those in *Lemma* 2 and Section 3.2.1 to extract the damping terms and obtain weighted $C^{1/2}$ and $L^{\infty }$ estimates of Eq. **12** up to a finite-rank operator Eq. **25**. To close nonlinear estimates with *ε* not extremely small (see constraint Eq. **5**), we aim to obtain a good bound on *λ* Eq. **5** for linear stability. For this purpose, we develop sharp $C^{1/2}$ estimates.

We have a key observation that the Hölder estimate of the nonlocal term with a sharp constant is related to an optimal transport problem. We focus on the main nonlocal term ${\tilde{u}}_{x}$. In the $C_{x}^{\alpha }$ estimate^#^ of $u_{x}\left(x,0\right)-u_{x}\left(z,0\right)$ with α∈(0,1), using the translation and scaling symmetries, we only need to estimate

[30] $$\begin{array}{c} \begin{array}{cc} S & =u_{x}\left(1/2,0\right)-u_{x}\left(-1/2,0\right)\\ & =-\frac{1}{\pi }P.V.\int_{R^{2}}k\left(s\right)\omega \left(s_{1},-s_{2}\right)ds, \end{array} \end{array}$$

in terms of ${\left||\omega |\right|}_{{\dot{C}}_{x}^{\alpha }}$, with ∫k(s)ds=0. In a simpler example where k(y)=δ(y−1)+δ(y−2)−δ(y−3)−δ(y−4), we have an optimal estimate

$$\begin{array}{cc} S & =|\int k(y)f(y)dy|\leq |f(2)-f(3)|+|f(1)-f(4)|\\ & \;\leq \left(1+3^{\alpha }\right){\left||f|\right|}_{{\dot{C}}^{1/2}}. \end{array}$$

It can be interpreted as moving the mass from 2 to 3 and 1 to 4 with the cost function ${\left|x-y\right|}^{\alpha }{\left||f|\right|}_{{\dot{C}}^{\alpha }}$. In general, an estimate of *S* using ${\left||\omega |\right|}_{{\dot{C}}^{\alpha }}$ is equivalent to estimating the transportation cost of moving the positive region of k(y) with measure $k^{+}\left(y\right)dy$ to its negative region with measure $k^{-}\left(y\right)dy$ with the concave cost function $c\left(x,y\right)={\left|x-y\right|}^{\alpha }$. Thus, to obtain a sharp estimate of *S* Eq. **30**, we seek a measurable transport map to obtain a cost as small as possible.

For more general 1D cases, we can derive the optimal estimate and the equation of the map:

Lemma 3. *[Transportation Lemma] Suppose there exists* c∈(a,b) *such that* *f* < *0 on* (a,c), *f* *> 0 on* (c,b), ${f|x-c|}^{\alpha }\in L_{\mathrm{loc}}^{1}$ *with* $\int_{a}^{b}f\left(x\right)dx=0$. *For* $\alpha \in \left(0,1\right),g\in C^{\alpha }\left(a,b\right)$, *we have*

$$\begin{array}{cc} |\int_{a}^{b}f\left(x\right)g\left(x\right)dx| & \leq \int_{c}^{b}{\left|f\left(x\right)||x-T\left(x\right)\right|}^{\alpha }dx\cdot {\left||g|\right|}_{{\dot{C}}^{\alpha }}, \end{array}$$

where T(x) solves $\int_{x}^{T(x)}f\left(s\right)ds=0$.

Note that we have the same optimal map T(x) for any α∈(0,1) since the cost function ${\left|x\right|}^{\alpha }$ is concave. The bound is given by some explicit integral depending on the map. To estimate Eq. **30**, we analyze the sign of $k(s_{1},s_{2})$ for each fixed *s*_2_ and apply *Lemma* 3 horizontally to construct the transport map T(s) in two dimensions, which solves a cubic equation.^‖^ For any $x,z\in R_{+}^{2}$ with $x_{2}=z_{2}$, we can obtain

[31] $$\begin{array}{c} |u_{x}\left(x\right)-u_{x}\left(z\right)|\leq C_{*}{\left|x_{1}-z_{1}\right|}^{1/2}{\left[\omega \right]}_{C_{x}^{1/2}}, \end{array}$$

where ${\left[\cdot \right]}_{C_{x}^{1/2}}$ denotes the seminorm (see Footnote #). The explicit formula of $C_{*}$ with $C_{*}\leq 2.55$ and a stronger version of Eq. **31** were established in ref. 15, lemma 3.1.

We can develop similar sharp $C_{x}^{1/2},C_{y}^{1/2}$ estimates (see Footnote #) for other terms in ∇u using *Lemma* 3. Using the triangle inequality, we can combine the Hölder estimates in $R_{+}^{2}$. We refer the details of these estimates to ref. 15, section 3 and appendix B.

We can generalize unweighted Hölder estimates to weighted ones with the same constant independent of the weight up to some more regular terms depending on the weight, like the commutator $B_{C}$ in Eq. **22b**. Estimates of the more regular terms follow Section 4.3.

### 3.5. Main part in the energy.

Based on the previous analysis, the main part of the energy (corresponding to *E* in Eq. **5**) consists of

$$\begin{array}{cc} E_{M}\left(f\right) & =\max(\max_{i\leq 3}||f_{i}\varphi_{i}|{|}_{L^{\infty }},\max_{i\leq 3}||f_{i}\psi_{i}|{|}_{C_{g_{i}}^{1/2}}),\\ {||F||}_{C_{g_{i}}^{1/2}} & =||\left(F\left(x\right)-F\left(y\right)\right)g_{i}\left(x-y\right){\left|\right|}_{L^{\infty }\left(R_{+}^{2}\times R_{+}^{2}\right)}, \end{array}$$

for some singular weights $\varphi_{i},\psi_{i}$, where $g_{i}\left(h\right)≍{\left|h\right|}^{-1/2}$ is $-\frac{1}{2}$-homogeneous and $\left|\right|\cdot {\left|\right|}_{C_{g_{i}}^{1/2}}$ is a seminorm equivalent to the $C^{1/2}$ seminorm. We modify $g_{i}\left(h\right)$ to explore the anisotropy of the flow ${\overset{¯}{c}}_{l}x+\overset{¯}{u}$. Since the $C^{1/2}$ estimate is akin to taking a half derivative, we design *ψ*_*i*_ to be ${\left|x\right|}^{1/2}$ more regular than *φ*_*i*_ near *x* = 0 (Eq. **29**) so that the energy *E*_*M*_ is well defined for $\omega ,\eta ,\xi =O(|x{|}^{3})$ near *x* = 0. Additional norms are included in the energy to obtain sharper stability estimates. We refer the reader to the full energy formulation in ref. 15, section 2.2. The energy *E*_*M*_ is applied to control the first component of the perturbation, namely *W*_1_, as discussed in Section 3.5.1.

In the weighted energy estimates, we must carefully estimate the associated coefficients-such as ratios between weights-and control the relevant variables in appropriate functional spaces. These steps lead to rather technical estimates, as detailed in ref. 15.

#### 3.5.1. Finite-rank perturbation.

In Section 3.2.1, we introduced methods for establishing the stability of the linearized Eqs. **14** and **25** with a finite-rank perturbation $K_{n}$. In this section, we further develop the finite-rank perturbation framework to estimate $K_{n}$.

We can rewrite the linearized equation Eq. **12** for W=(ω,η,ξ) schematically as $\partial_{t}W=LW$. We decompose $W=W_{1}+W_{2}$ with *W*_*i*_ solving

[32a] $$\begin{array}{c} \begin{array}{cc} & \partial_{t}W_{1}=\left(L-K_{n}\right)W_{1},\\ & \partial_{t}W_{2}=LW_{2}+K_{n}W_{1}, \end{array} \end{array}$$

using initial data $\left(W_{1},W_{2}\right){|}_{t=0}=\left(W{|}_{t=0},0\right)$, where $K_{n}W_{1}=\sum_{i\leq n}a_{i}\left(W_{1}\right){\overset{¯}{g}}_{i}$ is some finite-rank operator with given functions ${\overset{¯}{g}}_{i}$. We design $K_{n}$ to approximate more regular nonlocal terms and to obtain exponential decay estimates of *W*_1_ in some weighted norm *X*. See Section 3.2.1. Since *W*_2_ has zero initial data, we represent it using Duhamel’s formula

[32b] $$\begin{array}{c} \begin{array}{cc} W_{2}\left(t\right) & =\int_{0}^{t}e^{L(t-s)}(K_{n}W_{1}\left(s\right))ds\\ & =\sum_{i\leq n}\int_{0}^{t}a_{i}\left(W_{1}\left(s\right)\right)e^{L(t-s)}{\overset{¯}{g}}_{i}ds. \end{array} \end{array}$$

The stability estimate for *W*_1_ implies that $a_{i}\left(W_{1}\left(s\right)\right)$ decays exponentially. If $e^{Ls}{\overset{¯}{g}}_{i}$ decays in the energy space *X* as s→∞, we can derive decay estimates for *W*_2_ in *X*. It is important to note that the decay of $|e^{Ls}{\overset{¯}{g}}_{i}{|}_{X}$ for large *s* is not only sufficient but also necessary for the stability of L in *X*. This approach-obtaining the stability of an operator perturbed by a finite-rank operator K by testing decay on the modes in K-is related to the Sherman–Morrison formula (37) in linear algebra, and the T(1) (38) and T(b) (39, 40) theorems in harmonic analysis.

Note that numerical evidence of linear stability of L defined in Eq. **12** with a spectral gap has been obtained by Liu (36). Thus, one would expect that $e^{Ls}{\overset{¯}{g}}_{i}$ decays for large *s*. Since we can design ${\overset{¯}{g}}_{i}$ and L is given, to verify the decay of $e^{Ls}{\overset{¯}{g}}_{i}$, we construct its space-time approximation by solving

[33] $$\begin{array}{c} \partial_{t}{g=Lg,\;g|}_{t=0}={\overset{¯}{g}}_{i}, \end{array}$$

numerically and using a numerical basis and a representation similar to Eq. **36**. By interpolating the numerical solution at discrete time *t*_*k*_ using a cubic polynomial in *t*, we construct an approximate space-time solution ${\hat{g}}_{i}\left(t,x\right)$. To track the numerical error in solving the PDE, we use a posteriori error estimate and introduce the residual operator

[34a] $$\begin{array}{c} \begin{array}{cc} & R\left(W_{1},t\right)=\sum_{i\leq n}(a_{i}\left(W_{1}\left(t\right)\right)\left({\hat{g}}_{i}\left(0,x\right)-{\overset{¯}{g}}_{i}\right)\\ & \;+\int_{0}^{t}a_{i}\left(W_{1}\left(t-s\right)\right)\left(\partial_{s}-L\right){\hat{g}}_{i}\left(s,x\right))ds), \end{array} \end{array}$$

and modify the representation of *W*_2_ as follows

[34b] $$\begin{array}{c} {\hat{W}}_{2}=\sum_{i\leq n}\int_{0}^{t}a_{i}\left(W_{1}\left(t-s\right)\right){\hat{g}}_{i}\left(s,x\right))ds. \end{array}$$

We modify the decomposition Eq. **32** as follows


[34c] $$\partial_{t}W_{1}=(L-K_{n})W_{1}-R(W_{1},t),$$

[34d] $$\partial_{t}{\hat{W}}_{2}=L{\hat{W}}_{2}+K_{n}W_{1}+R(W_{1},t),$$

where $W_{1}{{|}_{t=0}=W_{0},\;{\hat{W}}_{2}|}_{t=0}=0$. Note that ${\hat{W}}_{2}$ solves Eq. **34d** exactly since the residual operator captures all the errors. If the error $\left(\partial_{s}-L\right){\hat{g}}_{i}\left(s,x\right)$ and ${\hat{g}}_{i}\left(0,x\right)-{\overset{¯}{g}}_{i}$ are small, we can show that the norm of the residual operator is small in some suitable functional space by using a bootstrap argument: $||R\left(W_{1},t\right){\left|\right|}_{X}\leq \hat{\varepsilon }\underset{s\leq t}{\sup}\left|\right|W_{1}\left(s\right){\left|\right|}_{X},\;\hat{\varepsilon }\ll 1.$ We treat R as a small perturbation to the stable part $L-K_{n}$ in Eq. **34c** and establish the stability for *W*_1_.

### 3.6. Finite-Rank Operator in refs. 15 and 16.

We construct the finite-rank operator $K_{n}$ by approximating the commutator in Eq. **22b**), and **u** in $L_{\nabla u},L_{u}$ Eq. **12** similar to Eq. **23**. Since the integral in $B_{C}\left(f\right)\left(x_{\mathrm{ij}}\right)$ Eq. **22b** is singular, we replace $B_{C}\left(f\right)\left(x_{\mathrm{ij}}\right)$ in Eq. **23** by a nonsingular version of $B_{C}\left(f\right)\left(x_{\mathrm{ij}}\right)$, where we remove a small region near the singularity $y=x_{\mathrm{ij}}$ in the integral Eq. **22b**. This adjustment provides a better overall approximation. Moreover, we perform a low rank perturbation so that the vanishing order $W_{1}={O(|x|}^{3})$ is preserved at x=0 in Eq. **34c**, which allows us to choose a more singular weight for weighted estimates in Section 3.2.1, i.e., smaller *α*_*f*_ in Eq. **26**; see, e.g., $\alpha_{\omega ,\eta ,\xi }=\left(-2.9,-3,-3\right)$ in Eq. **29**. See more details on the constructions in sections 4.2 and 4.3 in ref. 15.

### 3.7. Anisotropic Flow.

Due to the flow direction of $\overset{¯}{u}$, the outflow property Eq. **13c** is much stronger in the *y*-direction than in the *x*-direction. This allows us to obtain sharper stability estimates in $R_{+}^{2}$ away from the boundary and better control of *ξ* compared to *η* (see the damping terms in Eqs. **28a** and **28b**. Thus, we can focus the stability analysis on the coupling between *ω* and *η* in Eqs. **12a** and **12b**, formally neglecting the leading-order coupling to *ξ*. Moreover, the finite-rank perturbation method from Section 3.5.1 only needs to be applied near the boundary to approximate nonlocal terms such as $B_{C}$ in Eq. **22b**. We can also use a single cutoff function with O(1) support in the *y* (or *x*_2_) direction-i.e., setting *N* = 0 and *j* = 0 in the summation in Eq. **23**-which significantly reduces the rank *n* of $K_{n}$ in Eqs. **25** and **32a**.

#### 3.7.1. Nonlinear stability.

We treat the nonlinear terms Eq. **4** perturbatively and bound them by $CE^{2}$, where *E* is the energy. It is important to note that the stability constants, e.g., *λ*, *C* in Eq. **5**, are not sensitive to *ε*. Suppose that using a numerical method, we observe that the solution converges to a nontrivial steady state, and construct two approximate steady states ${\overset{¯}{W}}_{\varepsilon_{i}}=\left({\overset{¯}{\omega }}_{i},{\overset{¯}{\theta }}_{i},{\overset{¯}{c}}_{l,i},{\overset{¯}{c}}_{\omega ,i}\right)$ with a residual error *ε*_*i*_ (i=1,2). We expect that these profiles are *ε*_*i*_ close to the actual steady state $\left(\overset{¯}{\omega },\overset{¯}{\theta },{\overset{¯}{c}}_{l},{\overset{¯}{c}}_{\omega }\right)$ (which we do not know) in some suitable spaces.^**^ Then the constants in the stability estimates based on these two different profiles will be nearly identical. For example, we expect the following estimates for the terms ${\overset{¯}{u}}_{x}\eta$ in Eq. **12b**


$$|u_{x}\left({\overset{¯}{\omega }}_{1}\right)\eta -u_{x}\left({\overset{¯}{\omega }}_{2}\right)\eta |≲\left(\varepsilon_{1}+\varepsilon_{2}\right)\left|\eta \right|.$$

Thus, we can first perform stability estimates using a profile with a residual error that does not need to be extremely small and may not satisfy Eq. **5b**. After we determine *λ*, *C*, we refine the construction of the profile and verify the constraint Eq. **5b** a-posteriori.

#### 3.7.2. Computer-assisted estimates.

We summarize key steps involving computer assistance.

1. We construct the approximate profile and estimate the profile and the residual error using numerical methods. See Section 4.
2. We approximate the more regular nonlocal part, e.g., $B_{C}$, by a finite-rank operator $K_{n}$. To bound the approximation error-such as constants in the estimate of $B_{C}\left(f\right)-K_{\mathrm{MN}}\left(f\right)$ Eq. **24**-we evaluate certain integrals using numerical quadrature. See Section 4.3.
3. We track the constants in the stability estimates, which depend on the approximate profile, and compute sharp bounds with computer assistance.
4. For the finite-rank perturbation, we numerically construct the approximate solution to Eq. **33** using PDE solvers. See further discussion in Section 3.5.1.
5. We track discretization and round-off errors for rigorous numerics. Discretization error is analyzed using numerical analysis [Section 4.2, (16, section 3.6 and appendix C)], while round-off error is tracked via basic interval arithmetic (41). Both are treated as small perturbations in the energy estimates Eq. **5**.

In summary, we leverage the anisotropic structure (Section 3.5.1) to reduce the rank *n* of $K_{n}$ in Eqs. **25** and **32a**; control the approximation error in step 2 to improve *λ* and reduce *n*; track constants to refine bounds on *λ* and *C* in Eq. **5**; and estimate $K_{n}$ via the methods in Section 3.5.1. These steps, combined with analytic techniques from Sections 3.2.1–3.4.2, allow us to close the nonlinear stability estimate Eq. **5**.

## 4. Profile and Some Rigorous Numerics

In this section, we discuss constructing the approximate steady state to Eq. **9** and some rigorous numerics. The profile’s multidimensional nature requires solving a nonlocal, nonlinear PDE in 2D, posing an essential difficulty. To the best of our knowledge, the profile in the Hou–Luo scenario is the first truly multidimensional approximate self-similar blowup profile with sufficient regularity found for fluid equations.

### 4.1. Numerical Construction.

Due to the symmetry of ω(x,y),θ(x,y) in *x*, we only need to focus on $\left(x,y\right)\in R_{+}^{2}$. We represent the profile as follows

[35] $$\begin{array}{c} \begin{array}{cc} \overset{¯}{\omega } & ={\overset{¯}{\omega }}_{1}+{\overset{¯}{\omega }}_{2},\;{\overset{¯}{\omega }}_{1}=\chi \left(r\right)r^{\alpha }{\overset{¯}{g}}_{1}\left(\beta \right),\\ \overset{¯}{\theta } & ={\overset{¯}{\theta }}_{1}+{\overset{¯}{\theta }}_{2},\;{\overset{¯}{\theta }}_{1}=\chi \left(r\right)r^{1+2\alpha }{\overset{¯}{g}}_{2}\left(\beta \right), \end{array} \end{array}$$

where r=|(x,y)|,β= arctan(y/x)∈[0,π] denotes the polar coordinate in $R_{+}^{2}$, ${\overset{¯}{\omega }}_{2},{\overset{¯}{\theta }}_{2}$ capture the profile in the near field, and the semianalytic part ${\overset{¯}{\omega }}_{1},{\overset{¯}{\theta }}_{1}$ capture the far-field behavior. Here, *χ* is some cutoff function with *χ* = 0 near 0 and *χ* = 1 in the far-field. Functions ${\overset{¯}{g}}_{1}\left(\beta \right),{\overset{¯}{g}}_{2}\left(\beta \right)$ are the angular parts of the profiles $\overset{¯}{\omega },\overset{¯}{\theta }$ in the far-field, which cannot be determined a-priori. We represent $\left({\overset{¯}{\omega }}_{2},{\overset{¯}{\theta }}_{2}\right)$ in a domain ${\left[0,L\right]}^{2}$ with $L\approx 10^{13}$ using a piecewise 6-th order B-spline in *x* and *y*,

[36] $$\begin{array}{c} {\overset{¯}{\omega }}_{2}=\sum_{i,j}a_{i,j}B_{i}\left(x\right)B_{j}\left(y\right), \end{array}$$

where $B_{i}\left(x\right)$ is the B-spline basis (see ref. 16, appendix C.1). We represent $\overset{¯}{\theta }$ similarly. To construct $u=\nabla^{⊥}{\left(-\Delta \right)}^{-1}\omega$ Eq. **9**, we solve the Poisson equation −Δϕ=ω to obtain *ϕ* using a B-spline based finite element method and then obtain $u=\nabla^{⊥}\phi$.

To determine the profiles, we first solve Eqs. **9** and **10** numerically for a long enough time using the representation Eqs. **35** and **36** with time-dependent coefficients $a_{\mathrm{ij}}\left(t\right)$ and without the semianalytic part, i.e., setting ${\overset{¯}{\omega }}_{1}={\overset{¯}{\theta }}_{1}=0$, to obtain an approximate steady state $\left({\overset{¯}{\omega }}^{\left(2\right)},{\overset{¯}{\theta }}^{\left(2\right)},{\overset{¯}{c}}_{\omega }^{\left(1\right)},{\overset{¯}{c}}_{l}^{\left(1\right)}\right)$. We obtain the decay rate $\alpha ={\overset{¯}{c}}_{\omega }^{\left(1\right)}/{\overset{¯}{c}}_{l}^{\left(1\right)}$ in Eq. **35** by matching the slowest decaying parts in Eq. **9**

$$\begin{array}{cc} c_{l}r\partial_{r}\omega \left(r,\beta \right) & =c_{\omega }\omega +\theta_{x}+l.o.t.,\\ c_{l}r\partial_{r}\theta \left(r,\beta \right) & =(2c_{\omega }+c_{l})\theta +l.o.t., \end{array}$$

and use the far-field of $\left({\overset{¯}{\omega }}^{\left(2\right)},{\overset{¯}{\theta }}^{\left(2\right)}\right)$ and curve fitting to determine ${\overset{¯}{g}}_{1},{\overset{¯}{g}}_{2}$ in Eq. **35**. After we construct $\left({\overset{¯}{\omega }}_{1},{\overset{¯}{\theta }}_{1}\right)$, we fix it and refine the computation of $\left({\overset{¯}{\omega }}_{2},{\overset{¯}{\theta }}_{2},{\overset{¯}{c}}_{\omega },{\overset{¯}{c}}_{l}\right)$ by solving Eqs. **9** and **10** numerically using the representation Eqs. **35** and **36**. Using Eqs. **35** and **36**, we can evaluate the profile at any **x**. See more details in 15, section 7, (16, appendix C.1).

### 4.2. Rigorous Estimates of Profile and Error.

We use numerical analysis to estimate the profile and residual error. For example, to obtain a tight piecewise bound of a function *f* in $D_{\mathrm{ij}}\equiv \left[x_{i},x_{i+1}\right]\times \left[y_{j},y_{j+1}\right]$, we have

[37] $$\begin{array}{c} \begin{array}{cc} & \max_{x,y\in D_{\mathrm{ij}}}\left|f\left(x,y\right)\right|\leq \max_{i,j=1,2}|f(x_{i},y_{j}|\\ & \;+\frac{h_{1}^{2}}{8}\left|\right|\partial_{x}^{2}{f||}_{L^{\infty }\left(D_{\mathrm{ij}}\right)}+\frac{h_{2}^{2}}{8}\left|\right|\partial_{y}^{2}f{\left|\right|}_{L^{\infty }\left(D_{\mathrm{ij}}\right)}, \end{array} \end{array}$$

where $h_{1}=x_{i+1}-x_{i}$ and $h_{2}=y_{j+1}-y_{j}$. If *f* is a polynomial, e.g., ${\overset{¯}{\omega }}_{2}$ in Eq. **36**, the high-order derivatives vanish. Since we can evaluate the grid point values $f(x_{i},y_{j})$ using the representation formula, we can recursively use Eq. **37** to estimate the derivatives from high order to low order. For the semianalytic parts ${\overset{¯}{\omega }}_{1},{\overset{¯}{\theta }}_{1}$ in Eq. **35** and other functions, we first obtain a rough estimate on higher-order derivatives. Then we refine the piecewise bounds using Eq. **37** by subpartitioning the domain with smaller mesh *h*_*i*_.

For effective estimates without requiring too small *h*_*i*_, we use higher-order error estimates based on the Lagrange, Newton, and Hermite interpolations.

#### 4.2.1. Estimate in the far-field.

For very large |x|, we have ${\overset{¯}{\omega }}_{2}={\overset{¯}{\theta }}_{2}=0$, χ(r)=1, and $\overset{¯}{\omega }=r^{\alpha }{\overset{¯}{g}}_{1}$, $\overset{¯}{\theta }=r^{1+2\alpha }{\overset{¯}{g}}_{2}$ Eq. **35**. We estimate ${\overset{¯}{g}}_{i}\left(\beta \right)$ with β∈[0,π] using the 1D version of Eq. **37**. Since $r^{\gamma }$ is monotonic, its estimates are straightforward. We then obtain piecewise bounds for $r^{\gamma }{\overset{¯}{g}}_{i}\left(\beta \right)$, γ=α,1+2α, in polar coordinates, which can be transferred to the (x,y) coordinates via a covering argument. To verify inequalities in the far-field, e.g., Eq. **13c**, we factor out the decay rate $r^{k}$ for some *k* and verify inequalities in the angular variable.

### 4.3. Estimate Approximation Error.

We use a numerical quadrature to estimate the approximation error for the more regular terms.

We focus on a more regular nonlocal term **u** in Eq. **12** to illustrate the ideas. Let $\hat{u}\left(\omega \right)$ be the finite-rank approximation of **u** and *ρ* be a singular weight like *φ* defined in Eq. **19** in the energy estimates. We rewrite the error $\rho \left(x\right)(u\left(\omega \right)-\hat{u}\left(\omega \right))$ as an integral of *ω*: I(x)=ρ(x)∫K(x,y)ω(y)dy, and bound it using the weighted norm ${\left||\omega \rho |\right|}_{L^{\infty }}$. For example, the kernel associated with **u** is $K\left(z\right)=\frac{1}{2\pi }\nabla^{⊥}\log\left|z\right|$. To obtain a sharp estimate, we symmetrize the integral and estimate $I\left(x\right)=\rho \left(x\right)\int_{y_{i}\geq 0}K^{\text{sym}}\left(x,y\right)\omega \left(y\right)dy$ with the symmetrized kernel $K^{\text{sym}}$. We have two singularities in I(x): the singularity of ρ(x) near x=0 and the singularity at x=y in the kernel K(x,y).

In the case without an approximation, we get $K^{\text{sym}}\left(\lambda x,\lambda y\right)=\lambda^{-1}K^{\text{sym}}\left(x,y\right)$ for the kernel of **u** with −1 homogeneity. Changing $y=\lambda \hat{y},x=\lambda \hat{x}$ gives

$$\begin{array}{cc} I(x) & =\rho \left(x\right)\int_{R_{++}^{2}}K^{\text{sym}}\left(x,y\right)\omega \left(y\right)dy\\ & =\rho \left(\lambda \hat{x}\right)\int_{R_{++}^{2}}K^{\text{sym}}\left(\lambda \hat{x},\lambda \hat{y}\right)\omega \left(\lambda \hat{y}\right)\lambda^{2}d\hat{y}\\ & =\lambda \rho_{\lambda }\left(\hat{x}\right)\int_{R_{++}^{2}}K^{\text{sym}}\left(\hat{x},\hat{y}\right)\omega_{\lambda }\left(\hat{y}\right)d\hat{y}, \end{array}$$

where $f_{\lambda }\left(x\right)=f\left(\lambda x\right)$. We bound it using ${\left||\omega \rho |\right|}_{L^{\infty }}$:

$$\begin{array}{cc} & \left|I\left(x\right)\right|\leq \lambda ||\omega_{\lambda }\rho_{\lambda }{\left|\right|}_{L^{\infty }}\rho_{\lambda }\left(\hat{x}\right)\int_{R_{++}^{2}}\left|K^{\text{sym}}\left(\hat{x},\hat{y}\right)\right|\frac{1}{\rho_{\lambda }\left(\hat{y}\right)}d\hat{y}. \end{array}$$

Since $\left|\right|\omega_{\lambda }\rho_{\lambda }{\left|\right|}_{L^{\infty }}={\left||\omega \rho |\right|}_{L^{\infty }}$, it suffices to compute the rescaled integral. By choosing *λ* adaptive to **x**, e.g. $\lambda ≍\left|x|/\right|\hat{x}|$ with $|\hat{x}|\;≍1$, the singularity $\hat{y}=\hat{x}$ in the kernel is restricted to some finite domain away from the origin. Moreover, the integrand is locally integrable. Thus, we can design an adaptive mesh dense in the O(1) region to compute the integrals. In the case with an approximation term such as $\hat{u}\left(\omega \right)$, we can apply a similar rescaling argument to bound the integrals. For more details, see ref. 16, section 4.

## Data Availability

There are no data underlying this work.

## Footnotes

^*^In general, the blowup rate in front of *F* depends on the nonlinearity N(f) in Eq. **1**. Here, we adopt the blowup rate $\frac{1}{T-t}$, consistent with that of *ω* in Eq. **6**, and focus on this case for clarity.

^†^If *f* = *η* or *ξ*, the nonlocal terms ∇u depend on *ω* rather than *f*. Since the estimates can extend to the system of (ω,η,ξ) Eq. **12**, we assume that the nonlocal term only depends on *f* to simplify the model.

^‡^It is important that the constants $\overset{¯}{C},M_{k}$ are not sensitive to the residual error *ε* in Eq. **5a**. See more discussion in Section 3.7.1.

^§^Specifically, suppose that ${\left||B\left(f\varphi \right)|\right|}_{L^{\infty }}\leq C_{1}{\left||f\varphi |\right|}_{L^{\infty }}$, where *C*_1_ is independent of fφ. The above argument yields $C_{1}\left|\overset{¯}{b}\left(x\right)\right|+d\left(\varphi \right)\left(x\right)\leq -c<0$ uniformly in **x**. Thus, in the $L^{\infty }$ estimate-neglecting lower-order terms such as $B_{C}$-if the maximum of |fφ(·,t)| is attained at **x**, then |fφ(x,t)| decays in *t*, implying linear stability, i.e., *λ* > 0 in Eq. **5**. The full estimate is carried out in Lagrangian coordinates; see ref. 15, lemmas A.1 and A.2.

^¶^When φ(y) is very small, we still gain a factor |x−y| in $K_{C}=K_{B}\left(x-y\right)(1-\frac{\varphi (x)}{\varphi (y)})$. From Eq. **19** and *Lemma* 2, we have |∇φ(y)|≍|∇φ(z)| for any |y|≍|z|. If |x|≍|y|, then $|1-\frac{\varphi (x)}{\varphi (y)}|≲\frac{\left|\nabla \varphi (y)\right|}{\varphi (y)}\left|x-y\right|≲|x-y|\cdot \frac{1}{\left|y\right|}$. When |y|≍|x| is small, the singular factor ${\left|y\right|}^{-1}$ is absorbed by the coefficient $\overset{¯}{b}(x)$ in Eq. **22b**, since $\left|\overset{¯}{b}(x)|≲|x\right|$ by Eq. **17**. If |x|≪|y| or |y|≪|x|, then $K_{B}\left(x-y\right)$ is not singular. The integral of *K*_*C*_ is estimated using the methods in Section 4.3 by rescaling (x,y) to $\left(\hat{x},\hat{y}\right)$ with $|\hat{x|}≍1$ and partitioning the region for $\hat{y}$ into three regimes: $|\hat{y}\left|\ll \right|\hat{x}|$, $|\hat{y}\left|≍\right|\hat{x}|$, and $|\hat{x}\left|\ll \right|\hat{y}|$. See ref. 16, section 4 for details.

^#^We refer to $C_{x}^{\alpha }$ estimate as estimating ${\left|f\left(P\right)-f\left(Q\right)|/|P-Q\right|}^{\alpha }$ for $P,Q\in R_{+}^{2}$ with $P_{2}=Q_{2}$ but $P_{1}\neq Q_{1}$. We define $C_{y}^{\alpha }$ similarly and denote by ${\left[f\right]}_{C_{x}^{\alpha }},{\left[f\right]}_{C_{y}^{\alpha }}$ the seminorms.

^‖^For fixed x,z, we consider $K_{\Delta }\left(y\right)=K\left(x-y\right)-K\left(x-z\right)$ with kernel *K* associated with the velocity operator $\partial_{\mathrm{ij}}{\left(-\Delta \right)}^{-1}$. Using *Lemma* 3, we solve T(a) from $\int_{a}^{T(a)}K_{\Delta }\left(y\right)dy_{i}=0$ for *i* = 1 or *i* = 2, which reduces to a cubic equation of T(a) when $x_{1}=z_{1}$ or $x_{2}=z_{2}$. Moreover, using translation and scaling symmetries in the Hölder estimate, we can reduce the pairs (x,z) with $x_{i}=z_{i}$ depending on three parameters (e.g. $x_{1},z_{1},x_{2}$) to at most one parameter.

^**^This property holds if the linearized operator around the exact steady state is invertible.
